# Manual of Clinical Medicine: Special Pathology and Therapeutics, Regarded in a Clinical Point of View

**Published:** 1845-10

**Authors:** 


*Handbuch der Medicinischen Klinik: Die specielle Pathologie und Therapie von Klinischen Standpunkte aus bearbeitet.* Von Dr. Carl Canstatt, Königlich-bayerischem Gerichtsarzte und Mitgliede mehrerer gelehrter Gesellschaften. Zweite vermehrte Auflage. Erster Band. 8vo. S. 370. Erlangen, 1843.

*Manual of Clinical Medicine: Special Pathology and Therapeutics, regarded in a Clinical point of view.* By Dr. Charles Canstatt, &c. &c. Second augmented edition. Vol. 1, pp. 370. Erlangen, 1843.

The volume before us comprises what Dr. Canstatt terms the "Morphological part of Clinical Medicine," the elementary forms of disease. In other words, it is a treatise on general pathology; investigating in turn—Hypertrophy, Atrophy, Plethora, Anæmia, Chlorosis, Congestion, Hyperæmia, Inflammation, Hemorrhages, Blood diseases, Anomalies of Secretion, Dropsy, Bright's Disease, Pneumatosis, Polysarcia, Homoöplasia, (homologous changes of tissues,) Heteroplasia, (heterologous changes of tissue,) Carcinoma, Tuberculosis and Scrophulosis, Lithiasis, Formation of Entozoa, Softening, Induration, Fever, Neuroses, Neuralgia, Spasms, Anæsthesia, Acinesia, and Psychoses.

On all these topics the author exhibits that he is well read in modern authorities, and withal possessed of sufficient judgment to separate the good from the bad; whilst his own power of observation and reflection are greatly above mediocrity. There is much less, too, of fanciful speculation than usually exists in the productions of our German brethren.

